# Microbial Biotechnology‐2020

**DOI:** 10.1111/1751-7915.12403

**Published:** 2016-08-11

**Authors:** Kenneth Timmis, Juan Luis Ramos, Willem de Vos, Siegfried Vlaeminck, Auxi Prieto, Antoine Danchin, Willy Verstraete, Victor de Lorenzo

Microbial biotechnology is an exceptionally dynamic and exciting sector of the biomedical sciences that is unique in the breath and diversity of products and services it provides/will provide and to which it contributes, such as disease prevention and therapy, diagnostics, agriculture and horticulture, food provision and nutrition, energy production, production of chemicals and materials, water and waste treatment, recycling, acquisition of and adding value to natural resources, environmental monitoring, forensics, sustainable practices, etc. The development of rapid and affordable genomics technologies and accompanying bioinformatic tools, of systems and synthetic biology approaches, single cell techniques, and of high resolution analytical and imaging instruments, has provided new impulses to the field and opened new avenues of application, some of which, such as microbiome engineering, bioenergy and bioelectric applications, the use of microbial toxins for therapy and cosmetic applications, etc., promise to revolutionize our lives in a manner similar to that ushered in by the development of computers, the Internet and smart phones. The extent of present and future enrichment of human endeavour, prosperity and well‐being to be brought about by microbial biotechnology, as well as its contribution to solutions to fundamental problems we and planet Earth are facing – the Grand Challenges – is only now beginning to be appreciated.

The Editors and Friends of Microbial Biotechnology consider that this is an opportune moment to strategically analyse the immediate future of the field of microbial biotechnology. Luminaries in the field have therefore been invited to define where, in the context of Grand Challenges, we aspire to be in 2020, to articulate which obstacles lie in the way, and to suggest how these may be circumvented: in other words – to propose a road map for dealing with the obstacles and arriving at the goals set for 2020. These pieces make up this Special Issue entitled Microbial Biotechnology‐2020. We believe that this Special Issue will make interesting immediate reading for researchers in the field and serve as a useful guide over the next few years.

